# Challenges and opportunities of software automation discussed at the Yanqi-Lake Meeting

**DOI:** 10.1093/nsr/nwy145

**Published:** 2018-11-23

**Authors:** He Jiang

**Affiliations:** He Jiang is a professor of Dalian University of Technology, China

Scientists attending the Yanqi-Lake Meeting during this fall—a summit sponsored by the Chinese Academy of Sciences—discussed the challenges and opportunities of software automation in the big data era.

From 11 to 13 October 2018, nearly 40 distinguished scientists from Australia, Britain, Canada, China, Japan, Singapore and the USA gathered in Yanqi-Lake, Beijing, to exchange ideas on software automation, today and in the future.

Software automation—the process of generating software automatically based on formal or informal specifications—used to be a dream of computer scientists. Its purpose is not only to free developers from tedious programming for new features of software, but also free developers from the endless manual maintenance of evolving software under ever-changing environments. Software automation includes, but is not limited to, program synthesis, code completion, program transformation, code recommendation, program repair and software self-evolution. As an emerging and promising direction, software automation also implies a series of essential challenges, including vague and diverse requirements in open domains, complex software ecosystems and software technology stacks, and diversity in technical and business domains. It is even more challenging when software automation is required to handle nonfunctional requirements such as extendibility and safety.

Nowadays, the ‘big' software-engineering data, which are characterized with their volume, variety, velocity and veracity attributes, are driving this dream of software automation to come true. The participants at the Yanqi-Lake Meeting believe that some specific software-engineering tasks, such as bug fixing, will be able to be fully automated in the near future. Some participants even believe that we are about to witness computers gradually outperforming humans in programming in the coming decades. With software automation, a new type of pair programming may arise. That is, an intelligent assistant hidden within the Integrated Development Environment (IDE) is paired with a human developer at one workstation to perform daily development tasks. Devanbu, a professor from the University of California at Davis, said that the intelligent interaction between the IDE and human developers may be a breakthrough in the coming years.

All the scientists in the meeting agreed that ‘big' software-engineering data play a key role in software automation. Hence, in addition to the ‘big' data emerging in publicly available sources such as GitHub and Stack Overflow, scientists are also seeking more ways to manually label more software-engineering data. For example, with the support from the China Ministry of Science and Technology, Prof. Minghui Zhou from Peking University and Prof. Gang Yin from the National University of Defense Technology have set up a project to organize competitions among students on labeling open source code.

In this Yanqi-Lake Meeting, the scientists also discuss possible ways to classify the capability of software automation into a series of levels. A possible classification may be the automated generation of machine code (L1), automated generation of skeleton code and suggestion of the next line of code (L2), automated generation of code fragment (L3), automated generation of design structure (L4) and automated generation of the whole application based on requirements understanding (L5).

‘Software automation is promising with great challenges under the big data era. It is time to bring up researchers from various disciplines, including artificial intelligence, software engineering, and programming languages, to work together for software automation,' said Prof. Hong Mei, the chair of this Yanqi-Lake Meeting.

